# Impact of soil amendments on nitrous oxide emissions and the associated denitrifying communities in a semi-arid environment

**DOI:** 10.3389/fmicb.2022.905157

**Published:** 2022-08-17

**Authors:** Setor Kwami Fudjoe, Lingling Li, Yuji Jiang, Abdul-Rauf Malimanga Alhassan, Junhong Xie, Sumera Anwar, Linlin Wang, Lihua Xie

**Affiliations:** ^1^State Key Laboratory of Aridland Crop Science, Gansu Agricultural University, Lanzhou, China; ^2^College of Agronomy, Gansu Agricultural University, Lanzhou, China; ^3^State Key Laboratory of Soil and Sustainable Agriculture, Institute of Soil Science, Chinese Academy of Sciences, Nanjing, China; ^4^Department of Water Resources and Sustainable Development, The University of Environment and Sustainable Development, Somanya, Ghana; ^5^Institute of Molecular Biology and Biotechnology, The University of Lahore, Lahore, Pakistan

**Keywords:** denitrifiers, functional genes, keystone taxa, nitrous oxide emissions, potential denitrification activity

## Abstract

Denitrifying bacteria produce and utilize nitrous oxide (N_2_O), a potent greenhouse gas. However, there is little information on how organic fertilization treatments affect the denitrifying communities and N_2_O emissions in the semi-arid Loess Plateau. Here, we evaluated how the denitrifying communities are responsible for potential denitrification activity (PDA) and N_2_O emissions. A field experiment was conducted with five fertilization treatments, including no fertilization (CK), mineral fertilizer (MF), mineral fertilizer plus commercial organic fertilizer (MOF), commercial organic fertilizer (OFP), and maize straw (MSP). Our result showed that soil pH, soil organic carbon (SOC), and dissolved organic nitrogen (DON) were significantly increased under MSP treatment compared to MF treatment, while nitrate nitrogen (NO_3_^−^−N) followed the opposite trend. Organic fertilization treatments (MOF, OFP, and MSP treatments) significantly increased the abundance and diversity of *nirS*- and *nosZ*-harboring denitrifiers, and modified the community structure compared to CK treatment. The identified potential keystone taxa within the denitrifying bacterial networks belonged to the distinct genera. Denitrification potentials were significantly positively correlated with the abundance of *nirS*-harboring denitrifiers, rather than that of *nirK*- and *nosZ*-harboring denitrifiers. Random forest modeling and structural equation modeling consistently determined that the abundance, community composition, and network module I of *nirS*-harboring denitrifiers may contribute significantly to PDA and N_2_O emissions. Collectively, our findings highlight the ecological importance of the denitrifying communities in mediating denitrification potentials and the stimulatory impact of organic fertilization treatments on nitrogen dynamics in the semi-arid Loess Plateau.

## Introduction

Climate change mitigation is essential for carbon-neutral agriculture and food security (Zhang et al., 2020). Nitrous oxide (N_2_O) is a potential greenhouse gas, contributing to stratospheric ozone layer depletion (Cuello et al., 2015). Agricultural soils account for nearly 55% of global N_2_O emissions in 2015, and this percentage is projected to increase to 59% by 2030 (Hu et al., 2015). The transformations of nitrogenous compounds by soil denitrifying bacteria with excess fertilizer application is one of the main sources of N_2_O emissions (Tang et al., 2019).

During denitrification processes, nitrate (NO_3_^−^) is converted to nitrite (NO_2_^−^), nitric oxide (NO), N_2_O, and finally to dinitrogen (N_2_). These conversions are facilitated by nitrate reductase (NAR), nitrite reductase (NIR), nitric oxide reductase (NOR), and nitrous oxide reductase (NOS), respectively (Cui et al., 2016; Azziz et al., 2017). Nitrate reductase reduces NO_2_ to NO, a rate-limiting step of denitrification encoded by the copper-containing reductase gene (*nirK*) and cytochrome cd1 nitrite reductase gene (*nirS*) (Yoshida et al., 2009; Jang et al., 2018). Nitrous oxide reductase is encoded by *nosZ* gene, and functions in reducing N_2_O emissions (Yang et al., 2018). These functional genes are appropriate for studying the changes in *nirK*-, *nirS*-, and *nosZ*-harboring denitrifiers under different fertilization regimes (Yu et al., 2018; Chen et al., 2020).

Organic fertilization treatments considerably affect the abundance, diversity, and structure of the denitrifying communities, but their effects are not consistent (Ouyang et al., 2018). For instance, organic fertilizers have no significant effect on the abundance of denitrifiers (Yin et al., 2014), while the combined application of organic and mineral fertilizers increases the abundance of *nirS-* and *nosZ*-harboring denitrifiers and reduces N_2_O emissions (Zhang et al., 2015; Shi et al., 2019). The incorporation of crop straw increases soil organic carbon (SOC) and dissolved organic nitrogen (DON), and improves the abundance of *nirS*-harboring denitrifiers in alkaline soils (Huang et al., 2019). The changes in soil factors can differentially influence the *nirK*-, *nirS*-, and *nosZ*-harboring denitrifiers, thereby affecting their contributions to N_2_O emissions (Cui et al., 2016; Xiong et al., 2017). The network-based analytical approach is a powerful approach to investigate microbial associations and identify potential keystone taxa in the complex bacterial community (Barberán et al., 2012). Keystone taxa have highly linked functional features and greatly explain network structure. They are critical for minimizing community fragmentation and maintaining microbial community functioning (Williams et al., 2014; Herren and McMahon, 2018). Although the co-occurrence network of *nirK*-, *nirS*- and *nosZ*-harboring denitrifiers has recently been investigated in natural forest soil and arable black soil (Chen et al., 2019a; Yang et al., 2020b), little is known about the impact of organic fertilization treatments on denitrifying bacterial networks in the semi-arid loess plateau.

A growing body of studies has recently examined the effects of organic amendments on N_2_O emissions (Pang et al., 2019; Yang et al., 2020a). However, there is still limited knowledge on the mechanisms of the denitrifying communities in driving N_2_O emission in agroecosystems. It is critical to uncover the associations between the denitrifying bacterial communities and denitrification in the semi-arid loess plateau. For this purpose, we conducted a field experiment with the objectives to (1) evaluate soil properties, and the abundance, structure and co-occurrence network of *nirK*, *nirS*, and *nosZ*-harboring denitrifiers in response to fertilization treatments; (2) investigate the effect of organic fertilization treatments on the co-occurrence network of denitrifying communities; and (3) explore the relationships of soil properties and the denitrifying community with N_2_O emissions. We hypothesized that organic fertilization treatments would significantly improve the abundance and diversity of denitrifying communities, and alter the structure of denitrifying community by improving SOC and nutrient availability. We expected close links between the denitrifying community and N_2_O emissions across fertilization treatments.

## Materials and methods

### Experimental site and description of treatments

The field experiment was performed at the experimental station of Gansu Agricultural University in Dingxi, northwestern China (35°28′N, 104°44′E). The study area has a semi-arid environment with an annual frost-free period of 140 days and an average elevation of 2,000 m. The average annual rainfall in this area is 390 mm, with most rain falling between July and September. Calcaric cambisol is the aeolian soil type at this site, with a sandy loam texture. The tested soil has a pH value of 8.7, SOC content of 8.52 g kg^−1^, total nitrogen (TN) content of 0.93 g kg^−1^, and available phosphorus (AP) content of 15.3 mg kg^−1^. The maximum and minimum temperature of this site were 38°C in July and −22°C in January.

The experiment was started in 2012, including five treatments arranged in a completely randomized design with three replicates. The five fertilization treatments were: (i) no fertilization (CK); (ii) mineral fertilizer (MF) contained 200 kg N ha^−1^ of urea and 150 kg P_2_O_5_ ha^−1^ of triple superphosphate; (iii) mineral fertilizer plus commercial organic fertilizer (MOF) contained 3.03 t ha^−1^ of organic commercial fertilizer, 100 kg N ha^−1^ of urea, and 120 kg P_2_O_5_ ha^−1^ of triple superphosphate; (iv) Organic fertilizer (OFP) contained 6.06 t ha^−1^ of commercial organic fertilizer, and 90 kg P_2_O_5_ ha^−1^ of triple superphosphate; and (v) maize straw (MSP) contained 28.5 t ha^−1^ combined with triple superphosphate of 36 kg ha^−1^. In the spring, all fertilizers were spread evenly on the soil surface. The experimental plots were 13 m long and 3.3 m wide, with alternating narrow (15 cm high × 40 cm wide) and wide (10 cm high × 70 cm wide) ridges (Supplementary Figure S1). All ridges were covered with plastic film to increase soil temperature, reduce evaporative losses, and promote plant productivity. The seeds of maize (cultivar Pioneer 335) were sown at a density of 52,500 plants ha^−1^ in late April and harvested in October. Weeding was done manually between sowing and harvesting.

### Soil sampling and analysis

Soil samples were collected using a soil auger (5 cm diameter) at the flowering stage of maize in 2019. Five soil cores (0–20 cm) were collected along a zigzag line in each plot, and carefully mixed to obtain a composite sample. Soil samples were placed on dry ice and immediately transported to the laboratory. The soil samples were sieved at 2 mm to remove stones and roots. Each soil sample was divided into two halves, one kept at 4°C for chemical analysis and the other at −80°C for molecular analysis.

Soil pH was measured with a glass electrode in a 1:2.5 soil/water solution. SOC was determined by the oxidation of organic C with potassium dichromate (Nelson and Sommers, 1996). TN was measured using the CN elemental analyzer (LECO, Stockport, United Kingdom). Ammonia nitrogen (NH_4_^+^ − N) and nitrate nitrogen (NO_3_^−^−N) were extracted with 2 M KCl, and determined using a flow injection auto analyzer (FLA star 5000 analyzers, Foss, Denmark; Bremner, 1996). Dissolved organic nitrogen (DON) was extracted by 0.5 M K_2_SO_4_ and detected using a Multi N/C 2100 analyzer (Analytik Jena, Germany; Ghani et al., 2003). Available phosphorus (AP) was determined by colorimetric methods and resin extraction with modification (Olsen et al., 1954). Soil water content was measured by drying at 105°C for 24 h (Lamptey et al., 2019).

### Measurement of potential denitrification activity

Frozen soil samples were incubated at 25°C for 3 days. Potential denitrification activity (PDA) was determined using the acetylene inhibition method (Philippot et al., 2011), and was expressed as the N rate of N_2_O production (ng N_2_O N g^−1^ dry soil h^−1^). Briefly, 25 g of soil was placed in 125 ml plasma flask, and 25 ml of solution containing 10 mM KNO_3_, 10 mM glucose, and 50 mM K_2_HPO_4_. Chloramphenicol (0.1 g l^−1^) was added to suppress new protein formation. The flasks were evacuated to produce anaerobic conditions and purged with a 90:10 He-C_2_H_2_ gas combination to reduce N_2_O reductase activity. Gas samples were obtained after 0, 15, 30, 45, and 60 min after mixing. N_2_O concentrations was detected by a gas chromatograph (Agilent GC-7890A) with an electron capture detector.

### Measurement of N_2_O fluxes

Static chamber and gas chromatography were used to measure N_2_O fluxes. Each closed container (38 cm × 35 cm × 36.5 cm) was designed with a completely opaque covered with a corrugated tin foil to limit the influence of radiant heat during gas sampling. N_2_O gas samples were collected using a plastic syringe during sampling periods (0, 10, and 20 min after chamber closure) and deposited in an airtight aluminum bag for each sampling period (Dalian Delin gas packing, China). N_2_O gas samples were collected at 15-day intervals from May to September in 2019. Gas chromatography (Agilent 7890A, United States) was used to analyze the collected gas samples with an electron capture detector.

N_2_O flux (*f*) was calculated using the protocol described by Jantalia et al. (2008): *f = ρ ×* (*V*/*A*) *×* (*C*/*t*) *×* [273/(273 *+ T*)], where f is the N_2_O flux (μg m^−2^ h^−1^); *ρ* is the N_2_O gas density (kg m^−3^) at standard temperature and pressure; *V* is the chamber volume (m^3^); *A* is the soil area covered by the chamber (m^2^); *T* is the temperature in the chamber (°C); Δ*C*/Δ*t* is the change in N_2_O concentration inside the chamber during a given time (μl l^−1^ h^−1^). The cumulative N_2_O emissions (kg ha^−1^) were calculated using the following equation (Yeboah et al., 2021): M = ∑ (F_N + 1_ + F_N_) × 0.5 × (T_N + 1_ − T_N_) × 24 × 10^−2^, where M is the cumulative N_2_O emissions during the measurement period (kg ha^−1^); F is N_2_O (in mg m^−2^ h^−1^); N and N + 1 are the sampling emissions from the previous and current sampling; T is the number of days since the initial sampling.

### DNA extraction and quantitative polymerase chain reaction

Total DNA was extracted and purified from 0.5 g of fresh soil using the HiPure Soil DNA Mini Kit (Magen, Guangzhou, China). The quantity and purity of DNA were determined using a spectrophotometer (Nanodrop, PeqLab, Germany). The quantitative polymerase chain reaction (qPCR) was performed to detect the copy numbers of *nirK*, *nirS*, and *nosZ* genes using an ABI7500 thermocycler equipment (Applied Biosystems, Foster City, CA, United States). The primers to amplify denitrification gene are listed in Supplementary Table S1. The 20-μl reaction mixture contained 10 μl of SYBR Premix Ex Taq (TaKaRa Biotechnology, Tokyo, Japan), 0.5 μl of each primer (10 mM), 1 μl of DNA template (1–10 ng), and 8 μl of double-distilled water. DNA template was replaced with RNase-free ultrapure water as a control. The standard curves were generated to calculate the absolute abundance of *nirK*, *nirS*, and *nosZ* genes. Plasmids extracted from clones containing any of the target genes (*nirK*, *nirS,* and *nosZ*) were diluted to produce a series of standard templates (10^2^–10^8^ copies). The amplification efficiencies and *r*^2^ were >90% and 0.99%, respectively.

### Sequencing and processing of functional gene amplicons

DNA sequencing was used to investigate the abundance, diversity, and community structure of *nirS*, *nirK,* and *nosZ*-harboring denitrifiers. A unique 7-bp barcode sequence was added to the forward primers. The concentration of purified products was determined using a TBS-380 fluorometer (Turner Biosystems, CA, United States). The diluted PCR were paired-end sequenced on an Illumina MiSeq sequencer (Shanghai Personal Biotechnology, Co., Ltd., Shanghai, China). Raw sequences were quality screened and low-quality sequences were identified using Quantitative Insights Into Microbial Ecology (QIIME; Caporaso et al., 2010). Usearch was used to screen the chimeric assembled sequences (Edgar et al., 2011). The remaining high-quality sequences were then checked for frameshifts using the FrameBot tool of the Ribosomal Database Project (RDP) FunGene Pipeline (Edgar and Flyvbjerg, 2015). Next, the blastn algorithm compared the bacterial *nirK*, *nirS*, and *nosZ* sequences to the non-redundant nucleotide database GenBank (nt) in the National Center for Biotechnology Information (NCBI). Finally, the operational taxonomic units (OTUs) of each sample were determined by clustering the sequences using the cluster database at high identity with tolerance (CD-HIT-EST) algorithm, which requires a minimum sequence identity of 90% (Li and Godzik, 2006). Alpha diversity indices (Shannon index and Chao1 richness) of each functional gene were calculated using R software (version 3.5.3).

### Statistical analysis

The analysis of variance (ANOVA) with Tukey’s HSD test at *p* < 0.05 and Pearson correlation were performed using SPSS 21.0 (SPSS Inc., Chicago, IL, United States). Redundancy analysis (RDA) was used to evaluate the effects of soil physicochemical properties on the denitrifying communities using “vegan” package in R.

Co-occurrence networks were used to identify the significant taxa associations in the *nirK*-, *nirS*-, and *nosZ*-harboring denitrifiers. The OTUs that occurred in all replicates of each treatment were retained for network analysis. Pearson correlations between all nodes were performed, with the correlation coefficient (*r*) was >0.7 or < −0.7 and *p* value was <0.05. We then computed permutation and bootstrap distributions to evaluate the valid of edges with 1,000 iterations. The network was laid out using the Fruchterman–Reingold algorithm *via* Gephi (version 0.9.2). We calculated topological properties of networks, including the number of nodes and edges, average clustering coefficient, average degree, average path length, closeness centrality, network centrality, and modularity. The OTUs with the higher degree and closeness centrality were considered as potential keystone taxa (Berry and Widder, 2014).

Random forest modeling was used to identify the important predictors of N_2_O emissions, including soil variables and the denitrifying bacterial communities (Liaw and Wiener, 2002). The predictor importance of the model was quantified by the “A3R” package (Fortmannroe, 2015), and the significance of each predictor was determined by the “rfPermute” package (Archer, 2020). Structural equation modeling (SEM) was used to examine the direct and indirect effect of soil properties and the denitrifying communities on PDA and N_2_O emission using AMOS 21.0. The data distribution was tested for normality before modeling. The chi-square test (χ^2^, *p* > 0.05), root mean square error of approximation (RMSEA), and goodness-of-fit index (GFI) were used to determine the model fitness (Sahoo, 2019).

## Results

### Soil properties, potential denitrification activity, and N_2_O emission

Our results showed that soil properties significantly (*p* < 0.05) changed among fertilization treatments (Table 1). Soil pH ranged from 8.32 to 8.66 across fertilization treatments, and was significantly (*p* < 0.05) lower under MF treatment than under CK and MSP treatments. The MSP and OFP treatments significantly (*p* < 0.05) increased SOC and DON compared to MF and CK treatments, and significantly increased TN and AP compared to CK treatment (Table 1). NO_3_^−^−N concentration ranged from 17.84 to 30.81 mg kg^−1^, and was significantly (*p* < 0.05) increased under MF treatment compared to OFP, MSP, and CK treatments. Soil water content (SWC) was significantly (*p* < 0.05) higher under MF, MOF, OFP, and MSP treatments than under CK treatment (Table 1).

**Table 1:** Soil physicochemical characteristics under different fertilization treatments.

	CK	MF	MOF	OFP	MSP
pH	8.66 ± 0.03^a^	8.32 ± 0.07^c^	8.44 ± 0.08^bc^	8.45 ± 0.05^bc^	8.54 ± 0.06^b^
TN (g kg^−1^)	0.85 ± 0.01^b^	0.93 ± 0.03^a^	0.94 ± 0.02^a^	0.98 ± 0.03^a^	0.99 ± 0.02^a^
SOC (g kg^−1^)	7.48 ± 0.18^c^	7.93 ± 0.07^c^	8.81 ± 0.33^b^	8.84 ± 0.20^b^	9.81 ± 0.19^a^
NO_3_^−^−N (mg kg^−1^)	17.84 ± 1.04^c^	30.81 ± 2.78^a^	28.40 ± 3.57^ab^	25.40 ± 1.41^b^	21.93 ± 1.81^bc^
NH_4_^+^ − N (mg kg^−1^)	15.33 ± 1.63^a^	16.07 ± 1.48^a^	14.87 ± 2.22^a^	15.81 ± 1.36^a^	16.53 ± 2.80^a^
AP (mg kg^−1^)	9.73 ± 1.41^c^	16.70 ± 1.57^ab^	18.32 ± 1.69^ab^	19.81 ± 0.72^a^	15.14 ± 0.97^b^
DON (mg kg^−1^)	10.89 ± 0.62^b^	12.42 ± 0.78^b^	11.89 ± 1.04^b^	17.78 ± 1.25^a^	18.48 ± 1.57^a^
SWC (%)	23.11 ± 1.13^b^	28.21 ± 2.10^b^	32.42 ± 2.46^a^	31.93 ± 1.24^a^	28.63 ± 1.91^b^

Values are expressed as mean with standard error. Different lowercase letters indicate significant differences based on Tukey’s HSD test (*p* < 0.05). TN, total nitrogen; SOC, soil organic carbon; NO_3_^−^−N, nitrate nitrogen; NH_4_^+^ − N, ammonia nitrogen; AP, available phosphorus; DON, dissolved organic nitrogen; SWC, soil water content. CK, No fertilization; MF, mineral fertilizer; MOF, mineral fertilizer plus commercial organic fertilizer; OFP, commercial organic fertilizer; MSP, maize straw.

**Figure 1:**
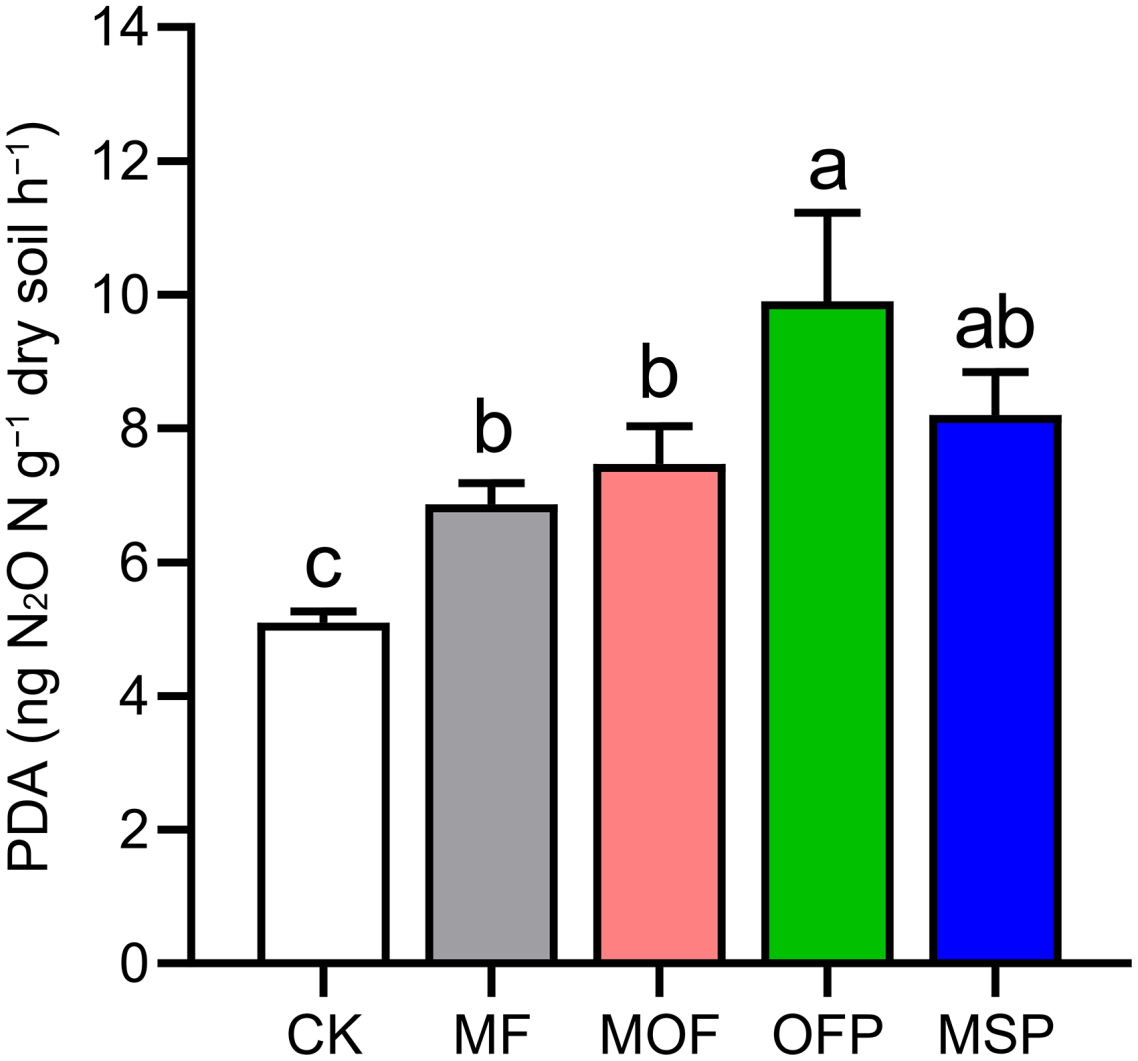
Potential denitrification activity (PDA) under different fertilization treatments. Bars (*n* = 3) with different lowercase letters indicate significant differences based on Tukey’s HSD test (*p* < 0.05). CK, No fertilization; MF, mineral fertilizer; MOF, mineral fertilizer plus commercial organic fertilizer; OFP, commercial organic fertilizer; MSP, maize straw.

**Figure 2:**
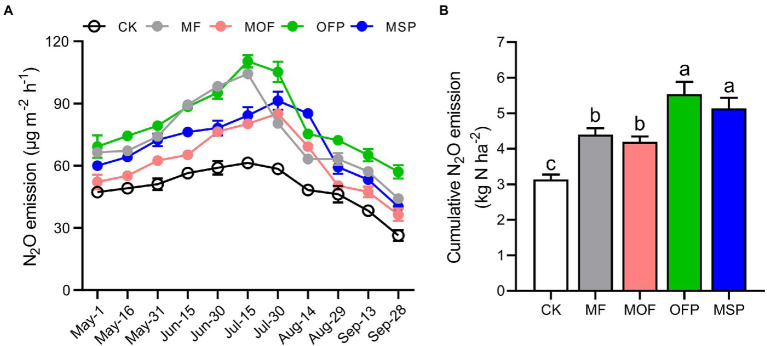
The changes in N_2_O emission flux **(A)** and cumulative N_2_O emission **(B)** among different fertilization treatments. Bars (*n* = 3) with different lowercase letters indicate significant differences based on Tukey’s HSD test (*p* < 0.05). CK, No fertilization; MF, mineral fertilizer; MOF, mineral fertilizer plus commercial organic fertilizer; OFP, commercial organic fertilizer; MSP, maize straw.

Potential denitrification activity (PDA) was significantly (*p* < 0.05) increased by 34.6%, 46.4%, 94.1%, and 60.8% under MF, MOF, OFP, and MSP treatments compared to CK treatment, respectively (Figure 1). Overall, N_2_O emissions were the highest in July and the lowest in September across fertilization treatments (Figure 2A). Furthermore, N_2_O emissions were significantly (*p* < 0.05) higher under OFP, MSP, and MF treatments than under MOF treatment. The cumulative N_2_O emissions under OFP, MSP, MF, and MOF treatments were significantly (*p* < 0.05) improved by 39.9%, 33.4%, 76.1%, and 63.4% compared to CK treatment, respectively (Figure 2B).

### Abundance, diversity, and composition of denitrifying communities

**Figure 3:**
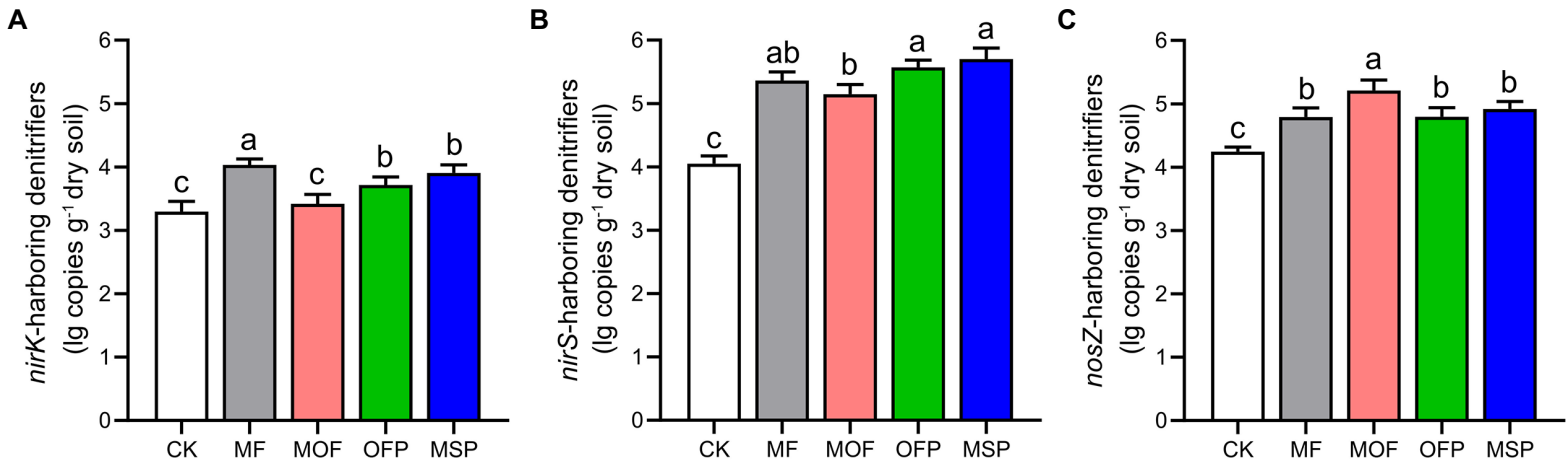
The copy numbers of *nirK* **(A)**, *nirS* **(B)**, and *nosZ* **(C)** genes under different fertilization treatments. Bars (*n* = 3) with different lowercase letters indicate significant differences based on Tukey’s HSD test (*p* < 0.05). CK, No fertilization; MF, mineral fertilizer; MOF, mineral fertilizer plus commercial organic fertilizer; OFP, commercial organic fertilizer; MSP, maize straw.

The abundance of *nirK*-harboring denitrifiers indicated by copy number of *nirK* gene was the highest under MF treatment and the lowest under CK treatment (Figure 3A). The abundance of *nirS*-harboring denitrifiers were significantly (*p* < 0.05) higher under OFP and MSP treatments than under CK and MOF treatments (Figure 3B). The abundance of *nosZ*-harboring denitrifiers under MOF treatment was significantly (*p* < 0.05) higher than those under CK, MF, OFP, and MSP treatments (Figure 3C). Shannon index and Chao1 richness of the denitrifying bacterial communities were significantly (*p* < 0.05) altered across fertilization treatments (Table 2), except for Shannon index of *nirK*-harboring denitrifiers. Shannon index and Chao1 richness of *nirS*- and *nosZ*-harboring denitrifiers were significantly (*p* < 0.05) enhanced under MOF and OFP treatments compared to CK and MF treatments. Shannon index and Chao1 richness of *nirK-*harboring denitrifiers were significantly (*p* < 0.05) higher under OFP treatment than under CK treatment.

**Table 2:** The diversity indices of *nirK*-, *nirS*-, and *nosZ*-harboring denitrifies under different fertilization treatments.

Denitrifiers	Treatments	Shannon index	Chao1 richness
	CK	4.69 ± 0.01^b^	1911 ± 60.8^b^
	MF	4.89 ± 0.06^ab^	2,156 ± 26.8^a^
*nirK*-harboring	MOF	4.94 ± 0.11^ab^	2,114 ± 10.3^ab^
denitrifiers	OFP	5.19 ± 0.15^a^	2,207 ± 40.9^a^
	MSP	4.92 ± 0.05^ab^	2095 ± 67.3^ab^
	*P* value	0.093	**0.036**
	CK	3.58 ± 0.08^c^	349 ± 18.6^b^
	MF	3.79 ± 0.03^bc^	441 ± 37.8^b^
*nirS*-harboring	MOF	4.10 ± 0.03^a^	853 ± 32.5^a^
denitrifiers	OFP	4.14 ± 0.06^a^	866 ± 24.6^a^
	MSP	3.83 ± 0.04^b^	770 ± 27.6^a^
	*P* value	**0.001**	**<0.001**
	CK	4.89 ± 0.11^d^	1,196 ± 52.9^c^
	MF	5.22 ± 0.03^c^	1,522 ± 46.8^b^
*nosZ*-harboring	MOF	5.59 ± 0.02^a^	2,139 ± 59.2^a^
denitrifiers	OFP	5.53 ± 0.06^ab^	1941 ± 41.9^a^
	MSP	5.37 ± 0.01^bc^	1,580 ± 54.3^b^
	*P* value	**<0.001**	**<0.001**

Values are expressed as mean with standard error. Different lowercase letters indicate significant differences based on Tukey’s HSD test (*p* < 0.05). Bold values denote significant effects. CK, No fertilization; MF, mineral fertilizer; MOF, mineral fertilizer plus commercial organic fertilizer; OFP, commercial organic fertilizer; MSP, maize straw.

**Figure 4:**
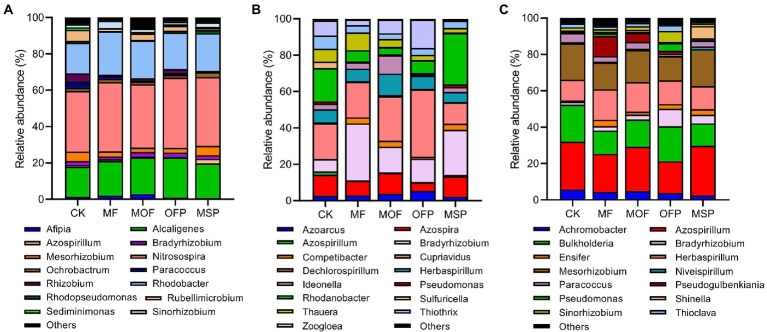
Taxonomic compositions of *nirK*- **(A)**, *nirS*- **(B)**, and *nosZ*-harboring **(C)** denitrifiers at the genus level under different fertilization treatments. CK, No fertilization; MF, mineral fertilizer; MOF, mineral fertilizer plus commercial organic fertilizer; OFP, commercial organic fertilizer; MSP, maize straw.

The *nirK*-harboring denitrifiers were dominated by *Nitrosospira* (36.6%), *Rhodobacter* (20.4%), *Alcaligenes* (19.4%), *Mesorhizobium* (3.6%), and *Rubellimicrobium* (3.0%; Figure 4A). The relative abundance of *Rhodobacter* and *Nitrosospira* was significantly (*p* < 0.05) higher under MF treatment than under CK and MOF treatments. However, *Alcaligenes* was significantly (*p* < 0.05) higher under CK and MSP treatment than under MOF and OFP treatments. Redundancy analysis (RDA) showed that soil pH (14.4%), SOC (13.2%), NO_3_^−^−N (13.0%), and TN (12.1%) significantly (*p* < 0.05) affected the structure of *nirK*-harboring denitrifier community (Supplementary Figure S2A; Supplementary Table S2). The *nirS*-harboring denitrifiers were dominated by *Cupriavidus* (22.5%), *Bradyrhizobium* (18.0%), *Rhodanobacter* (12.7%), *Azospira* (9.4%), *Herbaspirillum* (7.7%), and *Zoogloea* (7.1%; Figure 4B). The relative abundance of *Bradyrhizobium* and *Rhodanobacter* under MSP treatment was significantly (*p* < 0.05) higher than that under MOF and OFP treatments, while the relative abundance of *Cupriavidus* and *Zoogloea* followed the opposite trend. RDA indicated that NO_3_^−^−N (19.1%), SOC (16.6%), and pH (15.7%) significantly (*p* < 0.05) affected the structure of nirS-harboring denitrifier community (Supplementary Figure S2B; Supplementary Table S2). The *nosZ*-harboring denitrifiers were mainly comprised of *Azospirillum* (23.2%), *Mesorhizobium* (17.2%), *Burkholderia* (16.0%), and *Herbaspirillum* (14.0%; Figure 4C). The genera *Azospirillum* and *Mesorhizobium* were significantly (*p* < 0.05) higher under MOF and MSP treatments than under OFP treatment, whereas *Burkholderia* exhibited the inverse pattern. RDA revealed that SOC (20.5%), TN (18.2%), NO_3_^−^−N (15.6%), and pH (13.5%) significantly (*p* < 0.05) affected the structure of *nosZ*-harboring denitrifier community (Supplementary Figure S2C; Supplementary Table S2).

### Co-occurrence network of the denitrifying communities

The co-occurrence networks were constructed to investigate the critical modules within the denitrifying communities. There were more positive edges than negative edges in the networks of *nirK*-, *nirS*-, and *nosZ*-harboring denitrifiers (Supplementary Figure S3; Supplementary Table S3). The denitrifying bacterial networks were divided into distinct modules that were closely associated functional groups of taxa. Modules I, II, III, and IV of *nirK*-harboring denitrifier network comprised of 46, 43, 25, and 37 nodes, and 225, 178, 30, and 77 edges, respectively (Supplementary Table S3). Modules I, II, III, and IV of *nirS*-harboring denitrifier network consisted of 32, 26, 25, and 15 nodes, with 280, 76, 53, and 12 edges, respectively. There were 69, 41, 38, and 53 nodes, and 320, 181, 175, and 294 edges in the modules I, II, III, and IV of *nosZ*-harboring denitrifier network, respectively.

**Figure 5:**
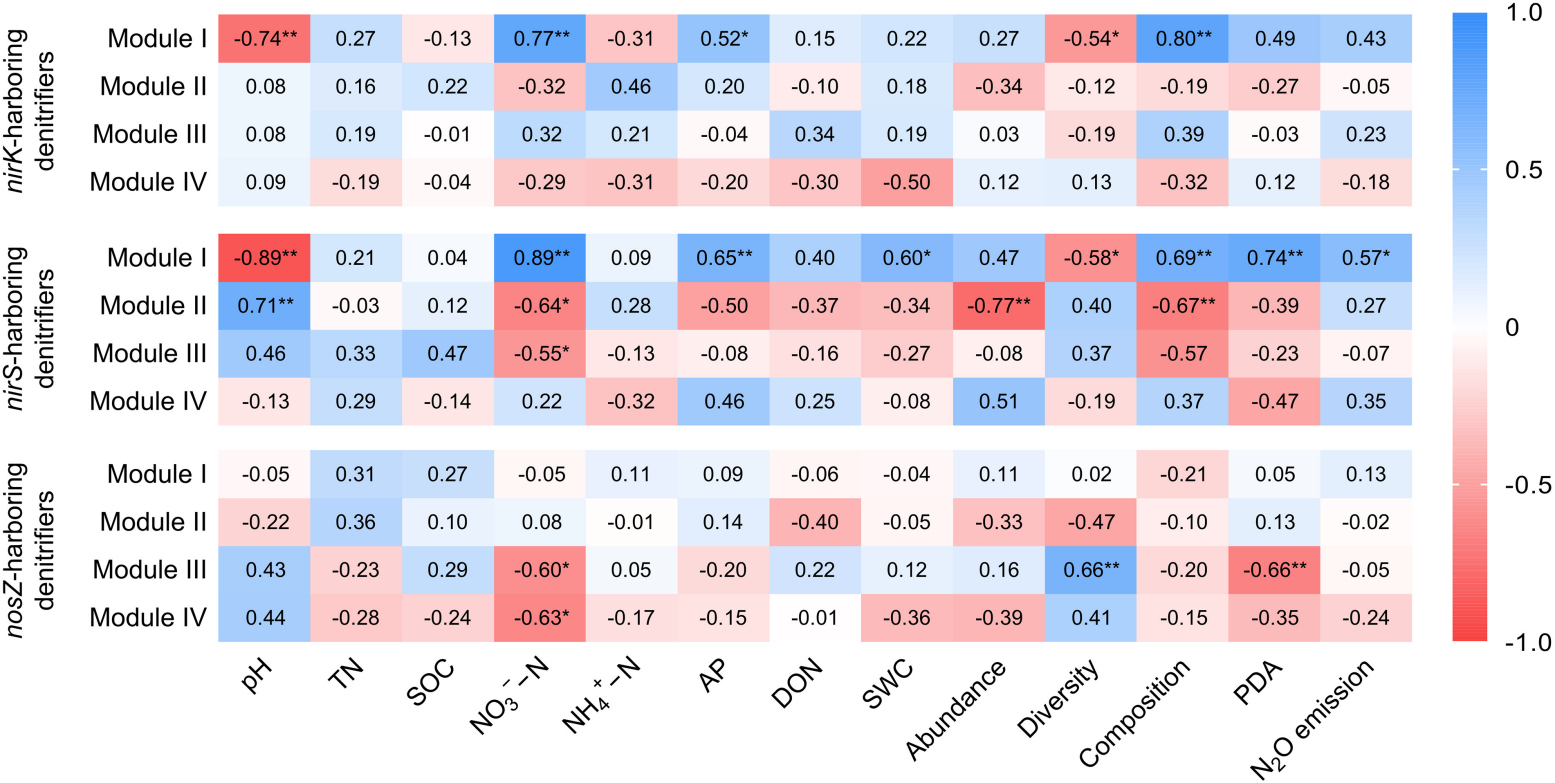
Correlation coefficients between module eigengenes, soil properties, the *nirK*-, *nirS*-, and *nosZ*-harboring denitrifiers, potential denitrification activity (PDA), N_2_O emission. The denitrifying communities are represented by abundance (the copy numbers of genes), diversity (Chao1 richness), and composition (first principal coordinates, PC1). Blue color indicates positive correlation, and red color indicates negative correlations. TN, total nitrogen; SOC, soil organic carbon; NO_3_^−^−N, nitrate nitrogen; NH_4_^+^ − N, ammonia nitrogen; AP, available phosphorus; DON, dissolved organic nitrogen; SWC, soil water content. ^*^p < 0.05 and ^**^p < 0.01.

Within the *nirK*-harboring denitrifier network, the genera *Alcaligenes*, *Nitrosospira*, and *Ochrobactrum* were identified as the potential keystone taxa in the module I (Supplementary Figure S3A). The module I of *nirK*-harboring denitrifier network showed significantly positive correlations with NO_3_^−^−N (*r* = 0.77, *p* < 0.01), AP (*r* = 0.52, *p* < 0.05), and community composition (*r* = 0.80, *p* < 0.01), but significantly negative correlations with pH (*r* = −0.74, *p* < 0.01) and diversity (*r* = −0.54, *p* < 0.05; Figure 5). The modules I in the *nirS*-harboring denitrifier network consisted of three keystone taxa affiliated with *Cupriavidus*, *Rhodanobacter*, and *Bradyrhizobium* (Supplementary Figure S3B). The module I of *nirS*-harboring denitrifier network exhibited positive relationships with NO_3_^−^−N (*r* = 0.89, *p* < 0.01), community composition (*r* = 0.69, *p* < 0.01), PDA (*r* = 0.74, *p* < 0.01), and N_2_O emissions (*r* = 0.57, *p* < 0.05), but negative relationships with pH (*r* = −0.89, *p* < 0.01; Figure 5). The module II of *nirS*-harboring denitrifier network were negatively correlated with NO_3_^−^−N (*r* = −0.64, *p* < 0.05), abundance (*r* = −0.77, *p* < 0.01), and community composition (*r* = −0.67, *p* < 0.01), but positively correlated with pH (*r* = 0.71, *p* < 0.01). The *nosZ*-harboring denitrifier network had six keystone taxa, belonging to *Azospirillum*, *Mesorhizobium*, *Burkholderia*, *Shinella*, *Ensifer*, and *Pseudomonas* (Supplementary Figure S3C). The modules III and IV of *nosZ*-harboring denitrifier network showed negative associations with NO_3_^−^−N (*r* = −0.64, *p* < 0.05 and *r* = −0.63, *p* < 0.05; Figure 5).

### Relationships between soil properties, the denitrifying bacterial communities, and N_2_O emissions

The abundance of *nirK*-harboring denitrifiers was positively associated with TN (*r* = 0.54, *p* < 0.05) and SOC (*r* = 0.74, *p* < 0.01), but negatively associated with N_2_O emissions (*r* = −0.62, *p* < 0.05). The abundance of *nirS*-harboring denitrifiers was positively correlated with pH (*r* = 0.53, *p* < 0.05), NO_3_^−^−N (*r* = 0.67, *p* < 0.01), SOC (*r* = 0.65, *p* < 0.05), DON (*r* = 0.51, *p* < 0.05), PDA (*r* = 0.54, *p* < 0.05), and N_2_O emissions (*r* = 0.57, *p* < 0.05). The abundance of *nosZ*-harboring denitrifiers was positively correlated with NO_3_^−^−N (*r* = 0.56, *p* < 0.05) and SWC (*r* = 0.68, *p* < 0.01), but negatively correlated with pH (*r* = −0.56, *p* < 0.05), SOC (*r* = −0.75, *p* < 0.01), and DON (*r* = −0.63, *p* < 0.05).

**Figure 6:**
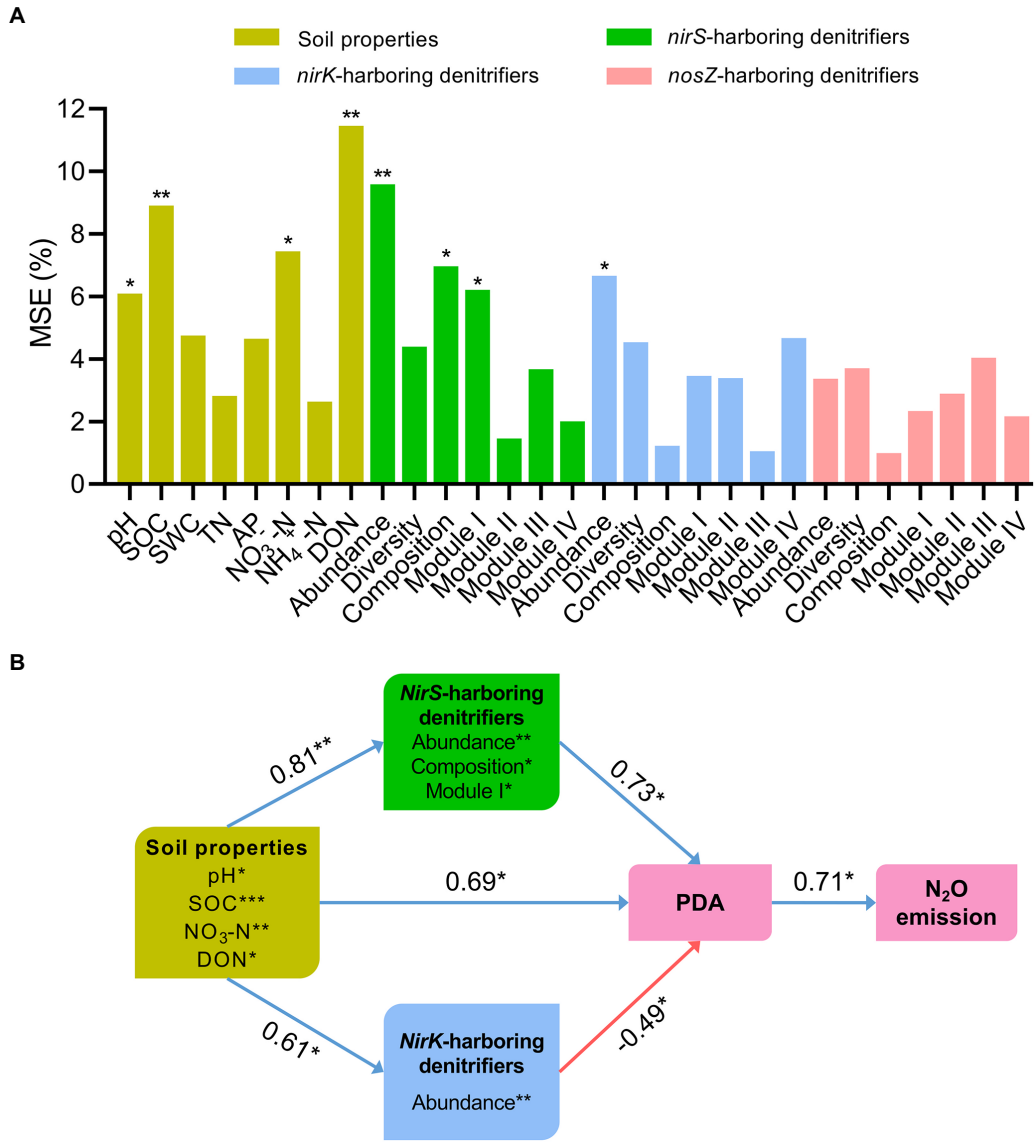
Random forest modeling was used to assess the main predictors of potential denitrification activity (PDA; **A**). Soil properties is represented by pH, soil organic carbon (SOC), soil water content (SWC), total nitrogen (TN), available phosphorus (AP), nitrate nitrogen (NO_3_^−^−N), ammonia nitrogen (NH_4_^+^ − N), and dissolved organic nitrogen (DON). The denitrifying communities are represented by abundance (the copy numbers of genes), diversity (Chao1 richness), composition (first principal coordinates, PC1) and network modules (module eigengenes). The significant predictors are further chosen to perform the structural equation modeling (SEM). SEM shows the direct and indirect effects of soil properties and the denitrifying communities on potential denitrification activity (PDA) and N_2_O emission across fertilization treatments **(B)**. Blue arrows indicate positive correlations, and red arrows indicate negative correlations. The numbers associated with arrows indicate the correlation coefficient. Widths of arrows indicate strength of significant standardized path coefficients. ^*^p < 0.05; ^**^p < 0.01.

Random forest modeling indicated that soil pH (6.1%, *p* < 0.05), SOC (8.9%, *p* < 0.01), NO_3_^−^−N (7.5%, *p* < 0.05), and DON (11.5%, *p* < 0.01) were the important abiotic variables predicting N_2_O emissions (Figure 6A). As for biotic variables, N_2_O emissions were significantly affected by the abundance (9.6%, *p* < 0.01), composition (6.9%, *p* < 0.05) and network module I (6.1%, *p* < 0.05) of *nirS*-harboring denitrifiers, and the abundance of *nirK*-harboring denitrifiers (6.5%, *p* < 0.05; Figure 6A). However, the *nosZ*-harboring denitrifiers exhibited no significant impact on N_2_O emissions. Structural equation modeling further showed that soil properties had significantly positive effects on the *nirS*- and *nirK*-harboring denitrifier communities (*r* = 0.81, *p* < 0.01 and *r* = 0.61, *p* < 0.05), and PDA (*r* = 0.69, *p* < 0.05; Figure 6B). Importantly, the *nirS*-harboring denitrifiers were positively correlated with PDA (*r* = 0.73, *p* < 0.05) through the abundance, community composition, and network module I (Figure 6B). However, the abundance of *nirK*-harboring denitrifiers was negatively associated with PDA (*r* = −0.49, *p* < 0.05).

## Discussion

### Soil properties and denitrifying communities in response to fertilization treatments

We found that OFP and MSP treatments considerably affected soil chemical properties compared to MF and CK treatments, including soil pH, SOC, DON, and NO_3_^−^−N. Organic fertilization has been widely proposed as alternative approach to solve the problems of excessive mineral fertilizer in sustainable farming systems (Seufert et al., 2012). Organic fertilizers (animal manures or maize straw) generally contains specifically high levels of organic matter content and micronutrients (Lamptey et al., 2019). The replacement of mineral fertilizer with organic fertilizers improves soil quality and physicochemical properties, as well as soil carbon and nitrogen stocks (Qiu et al., 2016). Correspondingly, the increase in SOC can provide readily available C sources for the microbial metabolism to improve DON immobilization (Cusack et al., 2011). However, OFP and MSP treatments significantly increased the potential denitrification activity, which was responsible for NO_3_^−^−N reduction relative to MF treatment.

In our study, inorganic fertilizer had a significantly positive effect on the abundance of *nirK*-harboring denitrifiers, while three organic fertilization treatments enhanced the abundance and diversity of *nirS-* and *nosZ-*harboring denitrifiers. Recent studies have reported that the *nirK*-harboring denitrifiers are more abundant than the *nirS*- and *nosZ*-harboring denitrifiers in different soils (Cui et al., 2016; Shi et al., 2019). However, our results showed that the abundance of *nirS*-harboring denitrifiers was significantly higher than that of *nosZ*- and *nirK*-harboring denitrifiers. In general, the variations of *nirS*-harboring denitrifiers in response to organic fertilization treatments can be largely responsible for the differences in SOC and N availability (Tao et al., 2018). The high level of SOC is considered to support the increasing abundance of *nirS*- and *nosZ*-harboring denitrifiers in soils treated with organic materials (Chen et al., 2019a). The *nirS*-harboring denitrifiers conduct the last step of denitrification, and are dependent on the changes in exogenous carbon and nutrient resource supply. Furthermore, soil pH can directly affect the cell growth and activities of *nirS*-harboring denitrifiers (Li et al., 2020b). The *nosZ*-harboring denitrifiers encode nitrous oxide reductase and promote denitrification to be carried out thoroughly. NO_3_^−^−N, an electron acceptor in denitrification, is strongly associated with the abundance and diversity of *nosZ*-harboring denitrifiers (Kastl et al., 2015). The predominance of *nosZ*-harboring denitrifiers under organic fertilization treatments facilitates the conversion of N_2_O to N_2_, thereby reducing N_2_O emissions (Shi et al., 2019).

Fertilization treatments lead to the changes in the bacterial life-history strategies, and contribute primarily to the variations in community structure (Fierer et al., 2012). Our results determined that the composition of microbial functional groups involved in denitrification responded significantly to fertilization treatments. The organic fertilization treatments presented the high abundance of *Cupriavidus* and *Zoogloea* in the *nirS*-harboring denitrifiers and *Mesorhizobium* and *Azospirillum* in the *nosZ*-harboring denitrifiers. The members of genus *Zoogloea* plays a key role in endogenous denitrification *via* nitrogen metabolism, promoting N_2_O emissions (Shen et al., 2020). In addition, the genera *Mesorhizobium* and *Azospirillum* promote diffusive transport of organic substrates, potentially enhancing the availability of organic N substrate for N-cycling microbial communities (Huang et al., 2020). As such, these dominant genera were considered to affect denitrification potentials and mediate N_2_O emissions under organic fertilization treatments.

### Microbial co-occurrence network and potential keystone taxa

Co-occurrence network analysis is often performed to investigate the species associations in the *nirK*, *nirS*, and *nosZ*-harboring denitrifier networks (Zhang et al., 2018). Overall, our results clearly showed that the number of edges and nodes was higher in the *nosZ*-harboring denitrifying network than in the *nirK*- and *nirS*-harboring denitrifier networks. There were more positive associations than negative ones in the networks, implying the intensive species cooperation and exchange events in the *nirK*-, *nirS*-, and *nosZ*-harboring denitrifiers. The high network centrality in the *nirS*-harboring denitrifier network showed better modularly organized in information transfer between bacteria, indicating their efficient performance within modules. Topological characteristics of the denitrifying bacterial networks can be further applied to statistically identify the modules (strong connecting structures among taxa) and potential keystone taxa based on their connections and central positions in the networks. The modules with higher modularity is likely to be more stable owing to stronger associations within functional groups (Maslov and Sneppen, 2002). The modules in denitrifying bacterial networks exhibited significant correlations with soil pH and NO_3_^−^−N, suggesting environmental changes might influence bacterial networks *via* these specific modules. We found that the potential keystone taxa within the modules of the denitrifying bacterial networks belonged to the distinct genera. The keystone taxa within the denitrifying bacterial networks may directly shape soil microbiome community assemblages with a disproportionate effect due to strong taxa interactions (Faust and Raes, 2012; Chen et al., 2019b). These keystone taxa form a close clustering with other taxa within the microbiome community, and contribute largely to network robustness (Zheng et al., 2022a). The keystone taxa may explain a large part of the network structure, and their removal causes considerable alterations in the stability and functioning of denitrifying bacterial community (Berry and Widder, 2014). However, caution is warranted when inferring the significant effects of keystone taxa on the denitrifying bacterial community. Further targeted culturomic approaches and empirical evidence are urgently needed to verify our findings on the contribution of potential keystone taxa to the entire networks.

### Denitrifying bacterial communities mediated N_2_O emissions

The denitrifying bacterial communities play crucial roles in the biogeochemical cycling of nitrogen through regulating potential denitrification activity (Domeignoz-Horta et al., 2018). We observed that the abundance, diversity and network module I of *nirS*-harboring denitrifiers were significantly correlated with PDA and N_2_O emission, rather than those of *nirK* and *nosZ*-harboring denitrifiers across five fertilization treatments. This result indicated that the denitrifying communities are not functionally similar under distinct soil environmental conditions. It is broadly accepted that niche differentiation has remarkable influence on the different behaviors and functional activities of denitrifying bacteria (Sun et al., 2017; Chen et al., 2020). Numerous literatures highlight their importance for N_2_O emissions, indicating by the positive relationship between the abundance of *nirS*-harboring denitrifiers and PDA (Ullah et al., 2020; Li et al., 2020a). Organic fertilization treatments provided a more balanced and sustainable nutrient resources for the diverse denitrifying bacterial populations, and the higher abundance of *nirS*-harboring denitrifiers increased N_2_O emissions. The *nirS*-harboring denitrifiers were proposed to be the numerical and functional dominance for a high denitrification efficiency over other denitrifying bacterial communities. Additionally, the module I with keystone taxa in the *nirS*-harboring denitrifier network was positively associated with PDA and N_2_O emissions. The keystone taxa have been commonly recognized as functional units that are of ecological importance in PDA and N dynamics irrespective of their abundance (Zhang et al., 2018; Zheng et al., 2022b). The species interactions mediated by potential keystone taxa in the *nirS*-harboring denitrifier network may improve positive abundance-functioning relationships. The large community size induced by keystone taxa contributes to the eventual promotion of denitrification potential and N_2_O fluxes in the natural field systems (Čuhel et al., 2010). Consequently, we suggested that the networks of the *nirS*-harboring denitrifiers could facilitate the community performance of denitrification at high levels of abundance.

## Conclusion

We observed that organic fertilization treatments significantly enhanced the abundance and diversity of *nirS*- and *nosZ*-harboring denitrifiers compared to CK treatment. Importantly, the abundance and the network module with keystone taxa of *nirS*-harboring denitrifiers exhibited exclusively positive relationships with denitrification potential and N_2_O emissions. Our study indicated the higher effect of *nirS*-harboring denitrifiers over *nirK*- and *nosZ*-harboring denitrifiers for denitrification and N_2_O fluxes. Taken together, this study provides insights into the response of the *nirS*-harboring denitrifiers to agricultural practices and the biotic mechanism behind positive affect on N_2_O emissions under organic fertilization treatments. As such, the deep understanding of mitigation measures for N_2_O emissions may pave the way for developing sustainable agroecosystems under a broad range of soil-climate scenarios.

## Data availability statement

The data of the *nirK*, *nirS*, and *nosZ* genes were deposited in the Sequence Read Archive of the NCBI database under accession number SRR18251436, SRR18481671, and SRR18481638, respectively.

## Author contributions

SF and LL: conceptualization and methodology. SF: validation, writing—original draft preparation, and visualization. JX and LW: resources. SF and LX: data curation. YJ, A-RA, SA, and LL: writing—review and editing. LL and YJ: supervision. JX: project administration. All authors have read and approved the content of the manuscript. All authors contributed to the article and approved the submitted version.

## Funding

The research was supported by the Education science and technology innovation project of Gansu Province (GSSYLXM-02), the National Natural Science Foundation of China (31761143004), the Youth Innovation Promotion Association of CAS (Y2021084), Science and technology Project of Jiangsu Province (BE2022394), and the Young Instructor Fund Project of Gansu Agricultural University (GAU-QDFC-2020-03).

## Conflict of interest

The authors declare that the research was conducted in the absence of any commercial or financial relationships that could be construed as a potential conflict of interest.

## Publisher’s note

All claims expressed in this article are solely those of the authors and do not necessarily represent those of their affiliated organizations, or those of the publisher, the editors and the reviewers. Any product that may be evaluated in this article, or claim that may be made by its manufacturer, is not guaranteed or endorsed by the publisher.

## Supplementary material

The Supplementary material for this article can be found online at: https://www.frontiersin.org/articles/10.3389/fmicb.2022.905157/full#supplementary-material
