# Supplementary material for: Impact of soil amendments on nitrous oxide emissions and the associated denitrifying communities in a semi-arid environment
Source: Front Microbiol. 2022 Aug 17;13:905157. doi: 10.3389/fmicb.2022.905157 (PMC9428159; doi:10.3389/fmicb.2022.905157)
Supplement: Supplementary file 1 [file Data_Sheet_1.docx]

**Supplementary Materials for**

**Impact of soil amendments on nitrous oxide emissions and the associated denitrifying communities in a semi-arid environment**

Setor Kwami Fudjoe, Lingling Li*, Yuji Jiang*, Abdul-Rauf Malimanga Alhassan, Junhong Xie, Sumera Anwar, Linlin Wang, Lihua Xie

*** Corresponding authors:**

Lingling Li lill@gsau.edu.cn

Yuji Jiang yjjiang@issas.ac.cn

**This PDF file includes:**

Figures S1 to S3

Tables S1 to S3


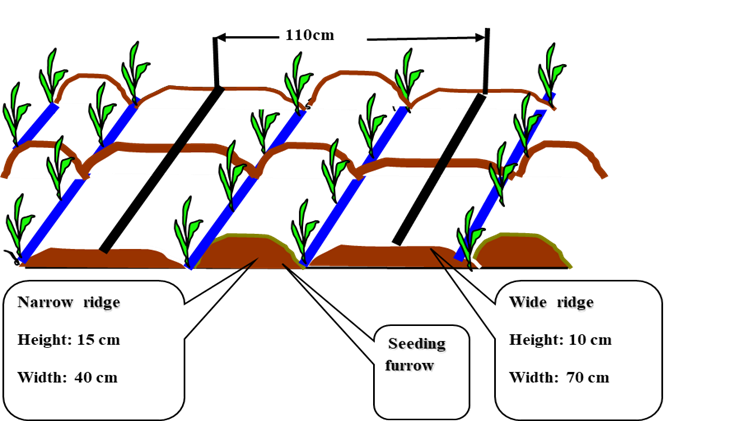


**FIGURE S1** An overview of the plot showing ridges.


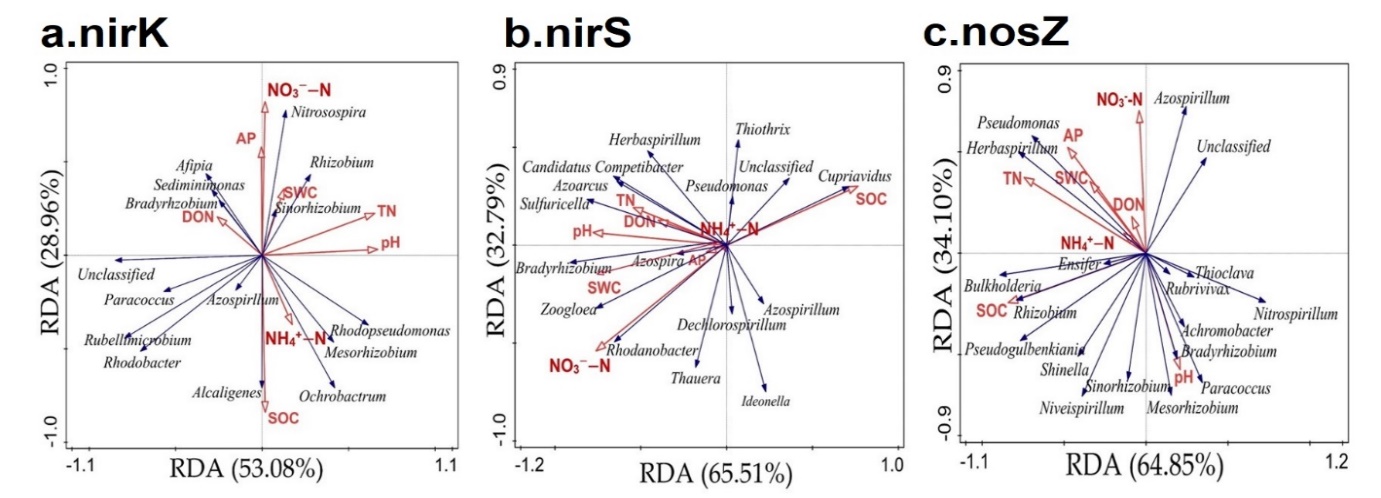


**FIGURE S2** Redundancy analysis indicates the associations between soil properties and the denitrifying communities. (**a**) *nirK­*-harboring denitrifiers. (**b**) *nirS*-harboring denitrifjkiers. (**c**) *nosZ*-harboring denitrifiers. TN, total nitrogen; SOC, soil organic carbon; NO_3_^−^−N, nitrate nitrogen; NH_4_^+^−N, ammonia nitrogen; AP, available phosphorus; DON, dissolved organic nitrogen; SWC, soil water content; PDA, potential denitrifying activity.


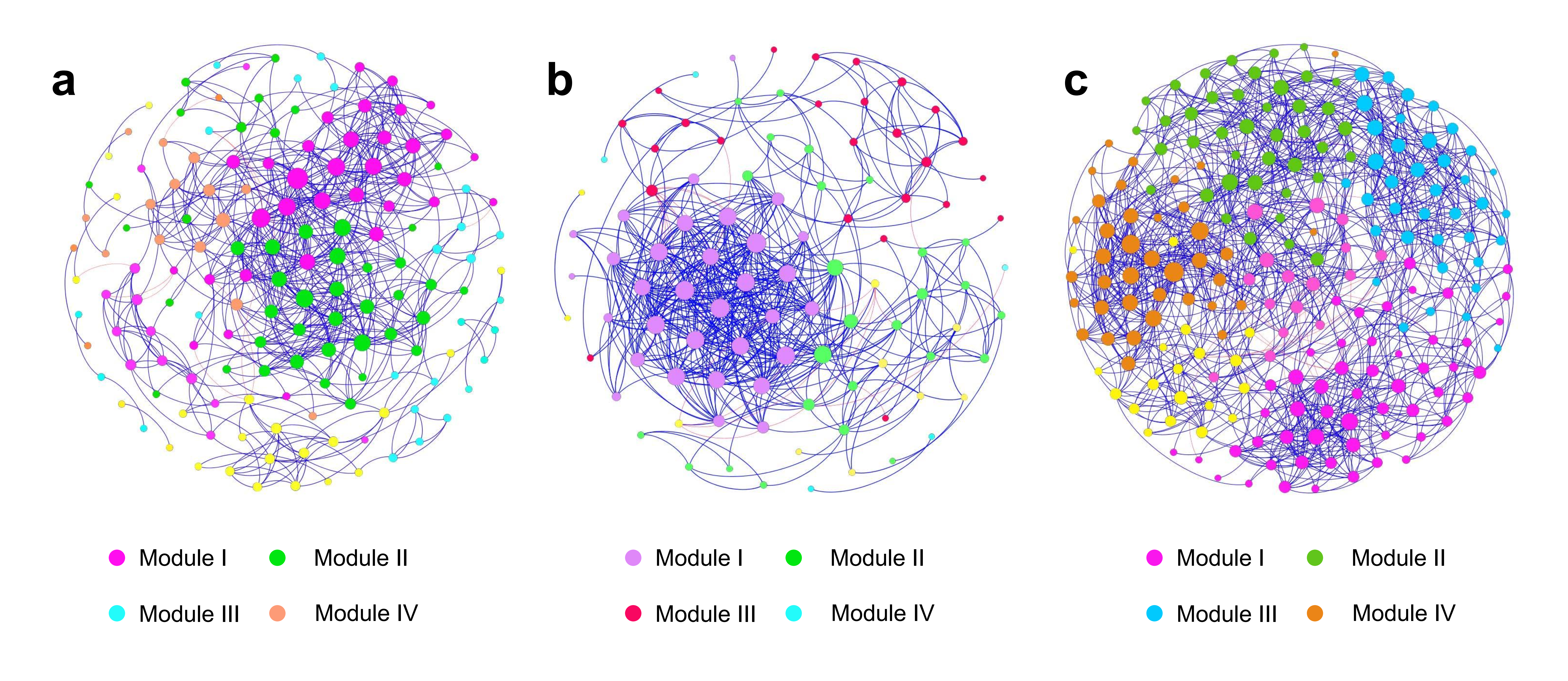


**FIGURE S3** Co-occurrence networks of the denitrifying communities across fertilization treatments.

(**a**) *nirK­*-harboring denitrifiers. (**b**) *nirS*-harboring denitrifiers. (**c**) *nosZ*-harboring denitrifiers. The modules I–IV in three networks were the four clusters of closely connected nodes. The size of each node is proportional to the number of connections (degree). The blue edges indicate positive correlations between two nodes, while red edges indicate negative correlations.

**TABLE S1** The specific primers for functional genes.

| Target gene | Primer sequence (5’-3’) | size (bp) | Reference |
| --- | --- | --- | --- |
| *nirK* | GGMATGGTKCCSTGGCA  GCCTCGATCAGRTTRTGG | 514 | Braker and Conrad, 2011 |
| *nirS* | GTSAACGTSAAGGARACSGG  GASTTCGGRTGSGTCTTGA | 473 | Throback et al., 2004 |
| *nosZ* | CCCGCTGCACACCRCCTTCGA  CGTCGCCSGAGATGTCGATCA | 300 | Chèneby et al., 1998 |

**References**

Braker, G., Conrad, R. (2011). Diversity, structure, and size of N_2_O-producing microbial communities in soils--what matters for their functioning? *Adv. Appl. Microbiol*. 75, 33−70. doi: 10.1016/B978-0-12-387046-9.00002-5

Chèneby, D., Hartmann, A., Hénault, C., Topp, E., Germon, J.C. (1998). Diversity of denitrifying microflora and ability to reduce N_2_O in two soils. *Biol*. *Fertil*. *Soils* 28, 19−26. doi: 10.1007/s003740050458

Throback, I.N., Enwall, K., Jarvis, A., Hallin, S. (2004). Reassessing PCR primers targeting *nirS*, *nirK* and *nosZ* genes for community surveys of denitrifying bacteria with DGGE. FEMS *Microbiol. Ecol*. 49, 401−417. doi: 10.1016/j.femsec.2004.04.011

**TABLE S2** Redundancy analysis between soil properties and the denitrifying communities.

| Denitrifiers | Environmental variables | Explain (%) | *F* | *P* |
| --- | --- | --- | --- | --- |
| *nirK*-harboring denitrifiers | pH | 14.4 | 2.2 | **0.020** |
|  | SOC | 13.2 | 2.0 | **0.026** |
|  | NO_3_^−^−N | 13.0 | 1.9 | **0.038** |
|  | TN | 12.1 | 1.8 | **0.048** |
|  | AP | 8.4 | 1.2 | 0.300 |
|  | NH_4_^+^−N | 7.3 | 1.1 | 0.440 |
|  | DON | 6.2 | 0.9 | 0.628 |
|  | SWC | 6.1 | 0.8 | 0.602 |
| *nirS*-harboring denitrifiers | NO_3_^−^−N | 19.1 | 3.1 | **0.002** |
|  | SOC | 16.6 | 2.6 | **0.010** |
|  | pH | 15.7 | 2.4 | **0.018** |
|  | SWC | 11.9 | 1.8 | 0.054 |
|  | TN | 11.7 | 1.3 | 0.062 |
|  | DON | 8.1 | 1.1 | 0.334 |
|  | AP | 7.1 | 1.0 | 0.432 |
|  | NH_4_^+^−N | 5.3 | 0.5 | 0.732 |
| *nosZ*-harboring denitrifiers | SOC | 20.5 | 3.4 | **0.002** |
|  | TN | 18.2 | 2.8 | **0.005** |
|  | NO_3_^−^−N | 15.6 | 2.4 | **0.010** |
|  | pH | 13.5 | 2.1 | **0.016** |
|  | AP | 11.2 | 1.6 | 0.102 |
|  | SWC | 8.7 | 1.2 | 0.260 |
|  | DON | 5.4 | 0.7 | 0.660 |
|  | NH_4_^+^−N | 2.5 | 0.4 | 0.968 |

Bold values denote significant effects. TN, total nitrogen; SOC, soil organic carbon; NO_3_^−^−N, nitrate nitrogen; NH_4_^+^−N, ammonia nitrogen; AP, available phosphorus; DON, dissolved organic nitrogen; SWC, soil water content.

**TABLE S3** Topological properties of *nirK*-, *nirS*-, and *nosZ*-harboring denitrifier networks.

| Denitrifiers | Module | I | II | III | IV |
| --- | --- | --- | --- | --- | --- |
| *nirK*-harboring denitrifiers | Node | 46 | 43 | 25 | 37 |
|  | Edge | 225 | 178 | 30 | 77 |
|  | Average clustering coefficient | 0.539 | 0.465 | 0.388 | 0.361 |
|  | Average degree | 11.370 | 10.441 | 2.720 | 5.430 |
|  | Average path length | 7.786 | 7.255 | 1.792 | 3.697 |
|  | Closeness centrality | 0.289 | 0.308 | 0.443 | 0.134 |
|  | Network centrality | 0.360 | 0.376 | 0.472 | 0.377 |
|  | Modularity | 0.599 | 0.524 | 0.328 | 0.347 |
| *nirS*-harboring denitrifiers | Node | 32 | 26 | 25 | 15 |
|  | Edge | 280 | 76 | 53 | 12 |
|  | Average clustering coefficient | 0.676 | 0.515 | 0.420 | 0.223 |
|  | Average degree | 13.031 | 8.500 | 4.880 | 2.350 |
|  | Average path length | 7.196 | 5.608 | 2.797 | 0.971 |
|  | Closeness centrality | 0.771 | 0.402 | 0.379 | 0.653 |
|  | Network centrality | 0.506 | 0.407 | 0.327 | 0.675 |
|  | Modularity | 0.680 | 0.429 | 0.501 | 0.212 |
| *nosZ*-harboring denitrifiers | Node | 69 | 41 | 38 | 53 |
|  | Edge | 320 | 181 | 175 | 294 |
|  | Average clustering coefficient | 0.379 | 0.381 | 0.369 | 0.416 |
|  | Average degree | 11.217 | 12.760 | 11.947 | 10.842 |
|  | Average path length | 7.495 | 8.668 | 8.011 | 7.304 |
|  | Closeness centrality | 0.489 | 0.392 | 0.544 | 0.639 |
|  | Network centrality | 0.382 | 0.407 | 0.389 | 0.382 |
|  | Modularity | 0.482 | 0.569 | 0.486 | 0.563 |
